# Growth and tolerance of formula with lactoferrin in infants through one year of age: double-blind, randomized, controlled trial

**DOI:** 10.1186/s12887-015-0488-3

**Published:** 2015-11-07

**Authors:** William H. Johnston, Claude Ashley, Michael Yeiser, Cheryl L. Harris, Suzanne I. Stolz, Jennifer L. Wampler, Anja Wittke, Timothy R. Cooper

**Affiliations:** Birmingham Pediatric Associates, 806 St Vincent’s Drive, Birmingham, AL 35205 USA; Southeastern Pediatric Associates, 364 Honeysuckle Road, Dothan, AL 36305 USA; Owensboro Pediatrics, 2200 E. Parrish Avenue, Owensboro, KY 42303 USA; Clinical Research, Department of Medical Affairs, Mead Johnson Nutrition, Evansville, IN 47721 USA; Global Research and Development, Mead Johnson Nutrition, Evansville, IN 47721 USA

## Abstract

**Background:**

Human milk provides necessary macronutrients (protein, carbohydrate, fat) required for infant nutrition. Lactoferrin (Lf), a multifunctional iron-binding protein predominant in human milk, shares similar protein sequence, structure, and bioactivity with bovine Lf (bLf). This large-scale pediatric nutrition study was designed to evaluate growth and tolerance in healthy infants who received study formulas with bLf at concentrations within the range of mature human milk.

**Methods:**

In this multi-center, double-blind, parallel-designed, gender-stratified prospective study 480 infants were randomized to receive a marketed routine cow’s milk-based infant formula (Control; *n* = 155) or one of two investigational formulas with bLf at 0.6 g/L (LF-0.6; *n* = 165) or 1.0 g/L (LF-1.0; *n* = 160) from 14–365 days of age. Investigational formulas also had a prebiotic blend of polydextrose (PDX) and galactooligosaccharides (GOS) and adjusted arachidonic acid (ARA). The primary outcome was weight growth rate from 14–120 days of age. Anthropometric measurements were taken at 14, 30, 60, 90, 120, 180, 275, and 365 days of age. Parental recall of formula intake, tolerance, and stool characteristics was collected at each time point. Medically-confirmed adverse events were collected throughout the study period.

**Results:**

There were no group differences in growth rate (g/day) from 14–120 days of age; 353 infants completed the study through 365 days of age (Control: 110; LF-0.6: 127; LF-1.0: 116). Few differences in growth, formula intake, and infant fussiness or gassiness were observed through 365 day of age. Group discontinuation rates and the overall group incidence of medically-confirmed adverse events were not significantly different. From 30 through 180 days of age, group differences in stool consistency (*P* < 0.005) were detected with softer stools for infants in the LF-0.6 and LF-1.0 groups versus Control.

**Conclusion:**

Compared to the Control, infants who received investigational formulas with bLf and the prebiotic blend of PDX and GOS experienced a softer stooling pattern similar to that reported in breastfed infants. This study demonstrated routine infant formulas with bLf, a blend of PDX and GOS, and adjusted ARA were safe, well-tolerated, and associated with normal growth when fed to healthy term infants through 365 days of age.

**Trial registration:**

ClinicalTrials.gov NCT01122654. Registered 10 May 2010.

## Background

Human milk provides the necessary protein, carbohydrate, and fat macronutrients required for infant nutritional needs. The protein component of cow’s milk-based infant formula is often patterned after mature human milk in which the ratio of whey and casein varies throughout lactation [1]. Lactoferrin (Lf), a multifunctional iron-binding protein predominant in the whey fraction (range, mature human milk: 0.44-4.4 g/L [2]), is involved in cellular proliferation and differentiation, iron status maintenance, host defense against microbial infection, anti-inflammatory activity and immune modulatory effects, and acts as a transcription factor (reviewed in [3]). Lf is synthesized by epithelial cells of the mammary glands and also present in other exocrine fluids [4]. Human Lf and bovine Lf (bLf) proteins share ~70 % sequence homology, are structurally similar, and in both in vitro and animal models have demonstrated comparable bioactivity (reviewed in [5]). Lf resists proteolysis in the infant gastrointestinal tract and uptake of both Lf and bLf has been demonstrated in vitro using an intestinal enterocyte model (reviewed in [3]). Minor amounts of bLF (≈30–485 mg/L) are available in cow’s milk [6], and therefore scarce in cow’s milk-based infant formula. A previous pilot study in healthy term infants demonstrated that addition of bLf (850 mg/L) to infant formula was well-tolerated, safe, and associated with a lower incidence of respiratory tract infections [7]. In children, dietary bLf lowered parasite colonization (1 g/day) [8] and reduced frequency and duration of vomiting and diarrhea (100 mg/day) [9]. Growth, safety, and a decrease in invasive fungal infections have been demonstrated in preterm infants who received bLf (100 mg/day) [10].

Human milk oligosaccharides (HMOs) are carbohydrates that comprise the third largest component in human milk (mature milk range, 5–15 g/L) following lactose and fat [11]. Over one hundred distinct HMOs have been identified, with composition differing between women and stage of lactation, which serve as a growth substrate for beneficial bacteria, inhibit pathogen adherence due to anti-adhesive properties, and modulate immune and intestinal epithelial cell responses [11]. Prebiotics, often used in infant formula to simulate the functionality of HMOs, are defined as “a selectively fermented ingredient that allows specific changes, both in the composition and/or activity in the gastrointestinal microbiota that confers benefits upon host well-being and health” [12]. Softer, looser stools are characteristic of both breastfed infants and infants who receive formula with (vs without) prebiotics [13–15]. In healthy term infants we previously demonstrated that cow’s milk-based infant formula with a prebiotic blend of polydextrose (PDX) and galactooligosacccharides (GOS) (1:1 ratio, 4 g/L) was well-tolerated, supported normal growth, promoted a stool consistency closer to that of breastfed infants [16] and produced a bifidogenic effect [17] and softer stools [16–19] when compared to infants who received a formula without PDX and GOS. This blend of PDX (mixture of complex, slowly fermented polysaccharides) and GOS (mixture of rapidly fermented oligosaccharides) covers the molecular weight range of most HMOs and meets the definition of a prebiotic used by the European Society for Paediatric Gastroenterology Hepatology and Nutrition (ESPGHAN) and the Food and Agriculture Organization of the United Nations (FAO) [20, 21].

The present study was designed to evaluate growth and tolerance in healthy term infants receiving infant formulas with bLf at 0.6 and 1.0 g/L with weight growth rate from 14–120 days of age as the primary outcome. Concentrations used for bLf were within the reported range for Lf in mature human milk. In addition, investigational formulas included the prebiotic PDX and GOS blend (4 g/L) as well as an adjustment in arachidonic acid (ARA) based on updated worldwide means for human milk docosahexaenoic acid (DHA) and ARA [22]. However, infant formulas may be provided up to 12 months of age even as complementary foods begin to be introduced. For this reason, we also assessed growth, tolerance, and adverse events for participants from 14 to approximately 365 days of age to assess the use of study formulas throughout the first year of life.

## Methods

### Study population

Healthy 12- to 16-day-old infants were recruited at 24 clinical sites in the United States. Eligible infants were singleton births at 37–42 weeks gestational age with birth weight ≥ 2500 g and solely formula-fed at least 24 h prior to randomization. Exclusion criteria included history of underlying disease or congenital malformation likely to interfere with normal growth and development or participant evaluation; signs of acute infection including fever, diarrhea, or antibiotic use; feeding difficulties or formula intolerance; weight at randomization <98 % of birth weight; large for gestational age born from a mother diabetic at childbirth; and immunodeficiency.

### Study design

In this multicenter, double-blind, randomized, controlled, parallel-group, prospective trial, participants were enrolled between July 2010 and January 2011. The study sponsor created a computer-generated, gender-stratified randomization schedule provided in sealed consecutively-numbered envelopes for each study site. Study formula was assigned by opening the next sequential envelope from the appropriate set at the study site. Study formulas, each designated by two unique codes known only to the sponsor, were dispensed to parents at each study visit prior to study completion or withdrawal. Participants were randomly assigned to receive one of three study formulas (isocaloric, 20 calories/fluid oz): a routine cow’s milk-based infant formula (Control; marketed Enfamil®, Mead Johnson Nutrition, Evansville, IN) or one of two investigational formulas with bLf (DMV International bv) at 0.6 g/L (LF-0.6) or 1.0 g/L (LF-1.0) from 14 days of age up to 365 days of age (Table 1). All study formulas provided DHA at 17 mg/100 kcal. ARA was provided at 34 mg/100 kcal in the Control and 25 mg/100 kcal in the investigational formulas. Each investigational formula also had 4 g/L (1:1 ratio) of a blend of PDX (Litesse® Two Polydextrose; Danisco) and GOS (Vivinal® GOS Galactooligosaccharide; Friesland Foods Domo).

**Table 1:** Nutrient composition per 100 kcal (20 Calories/fluid oz)

Nutrient	Study formula (target values)		
	Control	LF-0.6	LF-1.0
Total Protein, g	2.1	2.1^a^	2.1^b^
Total Fat, g	5.3	5.3	5.3
ARA, mg	34	25	25
DHA, mg	17	17	17
Total Carbohydrate, g	10.9	11.2^c^	11.2^c^
Vitamin A, IU	300	300	300
Vitamin D, IU	60	60	60
Vitamin E, IU	2	2	2
Vitamin K, mcg	9	9	9
Thiamin, mcg	80	80	80
Riboflavin, mcg	140	140	140
Vitamin B6, mcg	60	60	60
Vitamin B12, mcg	0.3	0.3	0.3
Niacin, mcg	1000	1000	1000
Folic Acid, mcg	16	16	16
Pantothenic Acid, mcg	500	500	500
Biotin, mcg	3	3	3
Vitamin C, mg	12	12	12
Choline, mg	24	24	24
Inositol, mg	6	6	6
Calcium, mg	78	78	78
Phosphorus, mg	43	43	43
Magnesium, mg	8	8	8
Iron, mg	1.8	1.8	1.8
Zinc, mg	1	1	1
Manganese, mcg	15	15	15
Copper, mcg	75	75	75
Iodine, mcg	15	15	15
Selenium, mcg	2.8	2.8	2.8
Sodium, mg	27	27	27
Potassium, mg	108	108	108
Chloride, mg	63	63	63

^a^with bLF, 0.6 g/L

^b^with bLF, 1.0 g/L

^c^with prebiotic blend of PDX and GOS (4 g/L)

## Ethics

Parents or guardians provided written informed consent prior to enrollment. The research protocol and informed consent forms observing the Declaration of Helsinki (including October 1996 amendment) were approved by the New England Institutional Review Board (IRB; Newton, MA); the University of Nebraska Medical Center, IRB, Office of Regulatory Affairs (Omaha, NE); Western IRB (Olympia, WA); and the University of Louisville IRB (Louisville, KY). The study complied with good clinical practices.

### Study objectives and outcomes

Anthropometric measures (body weight, length, and head circumference) were recorded at 14, 30, 60, 90, 120, 180, 275, and 365 days of age. At study enrollment, parents completed a baseline recall of tolerance (fussiness and gassiness), and stool characteristics (frequency and consistency) and provided information on participant’s history of breastfeeding (0, ≤7, or >7 days prior to study enrollment), family history of allergy in one or more relatives (including biological mother, father, sibling, and/or half-sibling) and exposure to smoking in the home. At all subsequent study visits, information on enrollment in daycare, exposure to smoking in the home and/or daycare, and 24-h recall of formula intake (fluid oz/day), tolerance (fussiness and gassiness), and stool characteristics (frequency and consistency) was collected. Responses were scaled from 0–3 for amount of gas (none, slight amount, moderate amount, excessive amount); 0–4 for fussiness (not fussy, slightly fussy, moderately fussy, very fussy, extremely fussy); and 1–5 for stool consistency (hard, formed, soft, unformed or seedy, watery). The primary outcome was weight growth rate from 14–120 days of age. Secondary outcomes included anthropometrics, tolerance measures, and medically-confirmed adverse events through 365 days of age. Adverse events were coded according to specific event (e.g. otitis media, colic, etc.) and the body system involved including: Body as a Whole; Cardiovascular; Eye, Ears, Nose, and Throat; Endocrine; Gastrointestinal; Metabolic and Nutrition; Musculoskeletal; Nervous; Respiratory; Skin; and Urogenital. Participants received exclusive study formula feeding through 120 days of age. Participants who continued in the study through 365 days of age were considered to complete the study even if study formula consumption discontinued or decreased to fewer than 2 feedings/day after 275 days of age (approximately 9-months-old).

### Statistical methods

The sample size was chosen to detect a clinically relevant difference of 3 g/day in weight gain from 14–120 days of age (80 % power; one-tailed). Assuming a standard deviation of 6 g/day for male and 5 g/day for female participants, approximately 78 males and 55 females were needed to enroll in each group with the expectation that 51 male and 36 female participants per study group would complete the study through 120 days of age. Analysis of variance (ANOVA) was used to assess growth rates in four pre-specified time intervals: from 14 to 30, 60, 90, or 120 days of age, calculated for each participant by linear regression of weight on age. Mean weight growth rates by gender for each investigational formula group were compared with the Control using one-tailed tests as outlined in guidance provided by the American Academy of Pediatrics (AAP) Task Force on Clinical Testing of Infant Formulas [23]. For all secondary outcomes, overall comparisons for the three formula groups were two-tailed. Unadjusted pairwise comparisons were performed if the overall test was statistically significant. All tests were conducted at α = 0.05. Achieved weight, length, and head circumference; length and head circumference growth rates; formula intake; and stool frequency were analyzed by ANOVA. Stool consistency, fussiness, and gas were analyzed using the Cochran-Mantel-Haenszel (CMH) row mean score test. Incidence of adverse events as well as incidence of allergic manifestations, gastrointestinal infections, respiratory infections, or any infection-related adverse events were analyzed using Fisher’s exact test. All analyses were conducted using SAS version 9.2 (Cary, NC).

## Results

### Participants

**Fig. 1:**
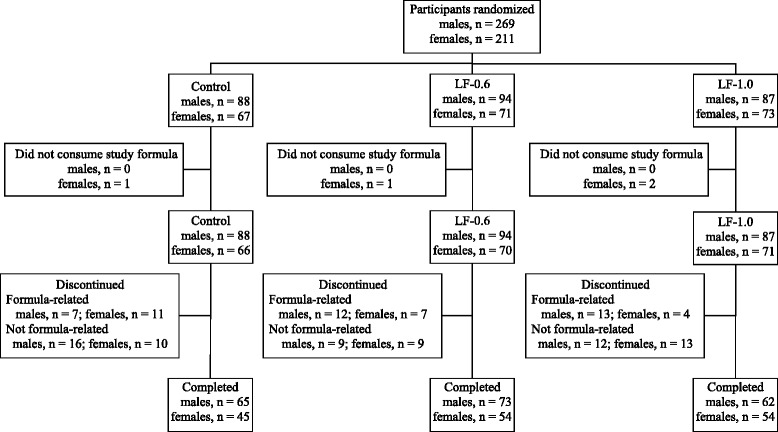
Flow of study participants

A total of 480 participants were enrolled and randomized (Control: 155; LF-0.6: 165; LF-1.0: 160). Participants who were randomized but consumed no study formula (Control: 1; LF-0.6: 1; LF-1.0: 2) were not included in subsequent analyses (Fig. 1). No differences in body weight, length, or head circumference were observed by gender among groups at study enrollment (Table 2). Birth anthropometric measures as well as gender, race, and ethnic distribution, history of breastfeeding, family history of allergy, and exposure to smoking in the home were also similar among groups (data not shown). No group differences from 30 to 365 days of age in daycare enrollment and exposure to smoking in the home and/or daycare were detected (data not shown). A total of 353 infants completed the study (Control: 110; LF-0.6: 127; LF-1.0: 116).

**Table 2:** Infant characteristics at study entry

	Study Group		
	Control	LF-0.6	LF-1.0
Total number of participants	154	164	158
Number of males/females	88/66	94/70	87/71
*males* ^a^			
Weight (g)	3662.4 ± 45.3	3649.2 ± 43.8	3704.8 ± 45.5
Length (cm)	52.3 ± 0.2	52.5 ± 0.2	52.7 ± 0.2
Head circumference (cm)	36.2 ± 0.1	36.2 ± 0.1	36.3 ± 0.1
*females* ^a^			
Weight (g)	3575.5 ± 45.5	3505.0 ± 44.2	3560.6 ± 43.9
Length (cm)	51.9 ± 0.2	51.5 ± 0.2	52.0 ± 0.2
Head circumference (cm)	35.7 ± 0.1	35.5 ± 0.1	35.7 ± 0.1

^a^Mean ± standard error (SE)

### Growth

**Fig. 2:**
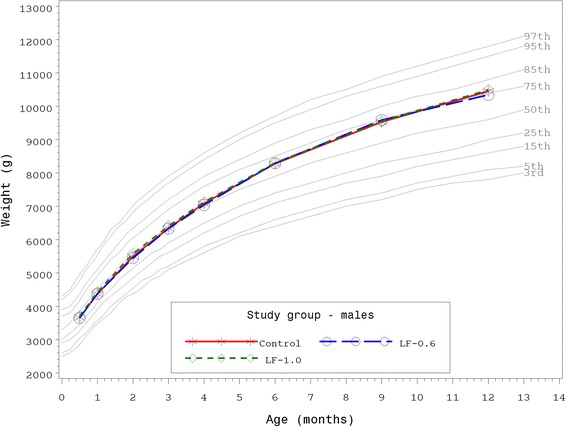
Mean achieved weight for male participants with World Health Organization (WHO) reference percentiles (3^rd^ to 97^th^) through 12 months (14 to 365 days) of age. Control, stars; LF-0.6, circles; LF-1.0, diamonds

**Fig. 3:**
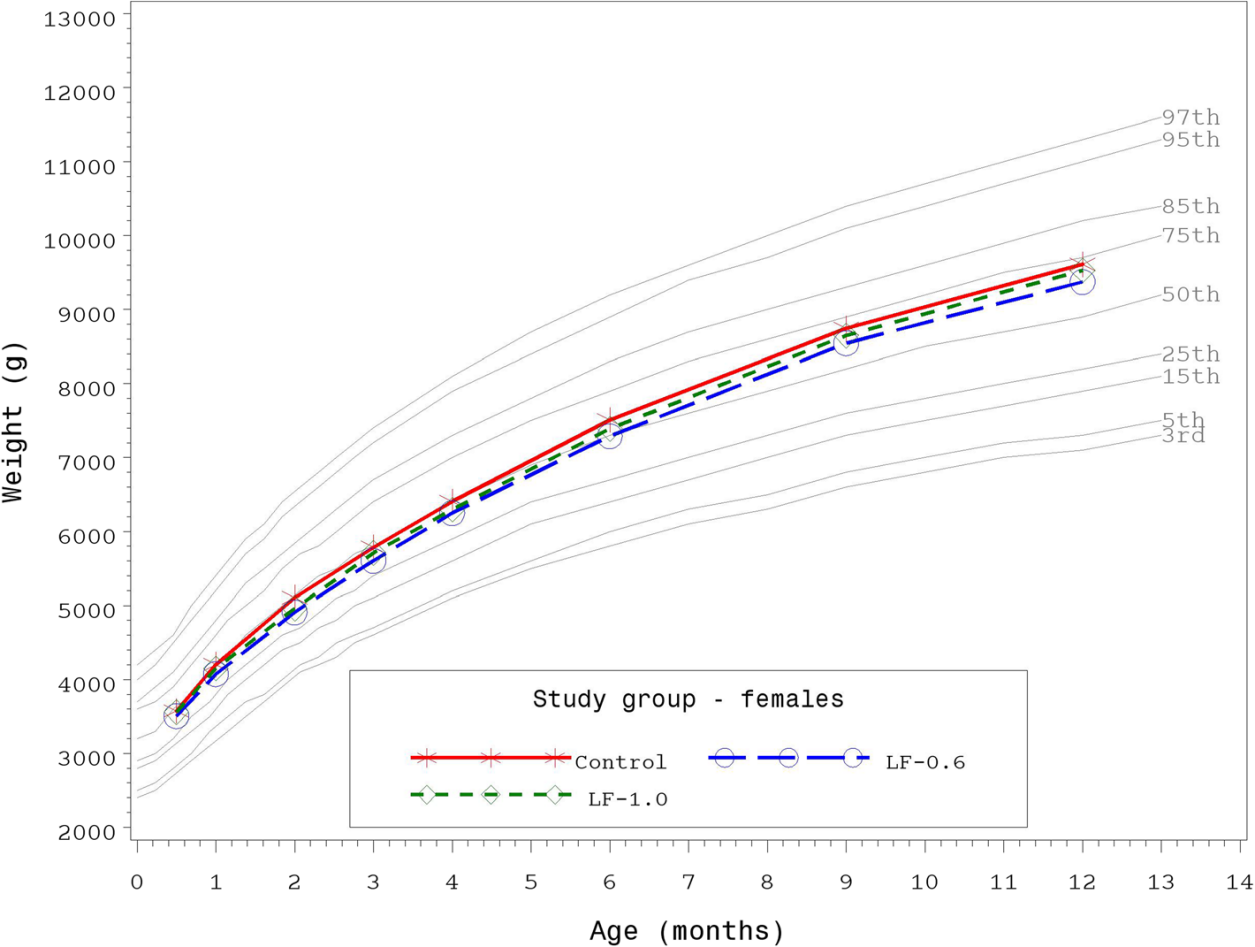
Mean achieved weight for female participants with World Health Organization (WHO) reference percentiles (3^rd^ to 97^th^) through 12 months (14 to 365 days) of age. Control, stars; LF-1.0, circles; LF-0.6, diamonds

Growth rates were analyzed from 14–365 days of age. As outlined in guidance provided by the AAP Task Force on Clinical Testing of Infant Formulas, rate of weight gain (g/day) is used as the most important parameter in clinical evaluation of infant formulas with differences of >3 g/day over a 3–4 month period considered clinically significant [23]. Consequently, no statistically significant group differences by gender in the primary outcome, weight growth rate from day 14–120, were detected (Table 3). No statistically significant differences were observed for weight, length, or head circumference growth rates by gender for any measured age range among study groups with the exception of lower weight growth rate for females in the LF-1.0 compared to the Control group from day 14–60 (29.7 ± 0.9 vs 32.4 ± 1.0 g/day; *P* < 0.05). This small difference within a single measured age range at less than 3 g/day was not considered clinically significant. In addition, no other statistically significant differences were observed for mean achieved weight, length, or head circumference at any measured time point up to 365 days of age. Mean achieved weight for males (Fig. 2) and females (Fig. 3) plotted on the WHO weight-for-age standard growth chart [24, 25] fell approximately within the 25^th^ and 75^th^ percentiles at all study time points.

**Table 3:** Weight, length, and head circumference growth rates from 14 days to 30, 60, 90, 120, 180, 275, and 365 days of age

			Growth rate^a^		
Gender	Day	Group (n)	Weight (g/day)	Length (cm/day)	Head circumference (cm/day)
male	30	Control (81)	45.1 ± 1.4	0.15 ± 0.009	0.09 ± 0.004
		LF-0.6 (92)	44.4 ± 1.3	0.14 ± 0.008	0.10 ± 0.004
		LF-1.0 (81)	44.3 ± 1.4	0.16 ± 0.009	0.10 ± 0.004
	60	Control (74)	39.0 ± 1.1	0.13 ± 0.004	0.07 ± 0.002
		LF-0.6 (86)	38.5 ± 1.0	0.13 ± 0.003	0.08 ± 0.002
		LF-1.0 (69)	39.6 ± 1.1	0.13 ± 0.004	0.07 ± 0.002
	90	Control (69)	35.1 ± 0.9	0.12 ± 0.002	0.06 ± 0.001
		LF-0.6 (82)	34.8 ± 0.8	0.12 ± 0.002	0.06 ± 0.001
		LF-1.0 (67)	35.4 ± 0.9	0.12 ± 0.002	0.06 ± 0.001
	120	Control (69)	31.8 ± 0.8	0.11 ± 0.002	0.06 ± 0.001
		LF-0.6 (80)	31.8 ± 0.7	0.11 ± 0.002	0.06 ± 0.001
		LF-1.0 (63)	31.9 ± 0.8	0.11 ± 0.002	0.06 ± 0.001
	180	Control (66)	27.5 ± 0.6	0.10 ± 0.001	0.05 ± 0.001
		LF-0.6 (78)	27.8 ± 0.6	0.09 ± 0.001	0.05 ± 0.001
		LF-1.0 (60)	27.4 ± 0.7	0.09 ± 0.002	0.05 ± 0.001
	275	Control (64)	22.5 ± 0.5	0.08 ± 0.001	0.04 ± 0.001
		LF-0.6 (73)	22.7 ± 0.4	0.08 ± 0.001	0.04 ± 0.001
		LF-1.0 (61)	22.3 ± 0.5	0.08 ± 0.001	0.04 ± 0.001
	365	Control (62)	19.1 ± 0.4	0.07 ± 0.001	0.03 ± 0.000
		LF-0.6 (68)	18.9 ± 0.3	0.07 ± 0.001	0.03 ± 0.000
		LF-1.0 (61)	19.1 ± 0.4	0.07 ± 0.001	0.03 ± 0.000
female	30	Control (60)	38.6 ± 1.5	0.12 ± 0.011	0.08 ± 0.005
		LF-0.6 (67)	35.7 ± 1.4	0.14 ± 0.010	0.08 ± 0.004
		LF-1.0 (67)	37.1 ± 1.4	0.13 ± 0.010	0.08 ± 0.004
	60	Control (54)	32.4 ± 1.0	0.12 ± 0.004	0.07 ± 0.002
		LF-0.6 (60)	30.6 ± 0.9	0.12 ± 0.004	0.06 ± 0.002
		LF-1.0 (59)	29.7 ± 0.9^b^	0.12 ± 0.004	0.06 ± 0.002
	90	Control (50)	28.1 ± 0.8	0.11 ± 0.003	0.06 ± 0.001
		LF-0.6 (58)	27.1 ± 0.8	0.10 ± 0.003	0.05 ± 0.001
		LF-1.0 (59)	27.5 ± 0.8	0.11 ± 0.003	0.06 ± 0.001
	120	Control (51)	26.2 ± 0.7	0.10 ± 0.002	0.05 ± 0.001
		LF-0.6 (58)	25.4 ± 0.7	0.10 ± 0.002	0.05 ± 0.001
		LF-1.0 (55)	25.5 ± 0.7	0.10 ± 0.002	0.05 ± 0.001
	180	Control (47)	23.4 ± 0.6	0.09 ± 0.002	0.04 ± 0.001
		LF-0.6 (57)	22.7 ± 0.5	0.09 ± 0.002	0.04 ± 0.001
		LF-1.0 (54)	22.9 ± 0.5	0.09 ± 0.002	0.04 ± 0.001
	275	Control (46)	19.7 ± 0.5	0.07 ± 0.001	0.03 ± 0.001
		LF-0.6 (55)	19.4 ± 0.4	0.07 ± 0.001	0.03 ± 0.001
		LF-1.0 (54)	19.5 ± 0.4	0.07 ± 0.001	0.03 ± 0.001
	365	Control (44)	16.9 ± 0.4	0.07 ± 0.001	0.03 ± 0.000
		LF-0.6 (53)	16.6 ± 0.4	0.06 ± 0.001	0.03 ± 0.000
		LF-1.0 (54)	16.9 ± 0.3	0.07 ± 0.001	0.03 ± 0.000

^a^Mean ± standard error (SE)

^b^Significantly lower than Control, *P* < 0.05, one-tailed test

### Tolerance

Parent-reported mean study formula intake (fl oz/day; data not shown) increased from day 30–120 for all groups by gender, indicating normal intake for LF-0.6 and LF-1.0 groups when compared to the Control for this time period (examples: females at day 30, 25.9–26.9 fl oz/day and day 120, 33.3–34.2 fl oz/day; males at day 30, 28.2–30.0 fl oz/day and day 120, 35.0–35.7 fl oz/day). Intake for female participants by group was similar at all time points assessed. Statistically significant group differences in intake were noted among males at days 180 (Control: 36.5 fl oz/day; LF-0.6: 31.9 fl /day; LF-1.0: 33.7 fl oz/day; Control vs LF-0.6, *P* < 0.05) and 275 only. However, by day 180, mean reported study formula intake began to decline in general for both male and female participants which could be expected as parents and caregivers likely begin to offer complementary foods to infants at approximately 4–6 months of age. Parent-reported gassiness and fussiness were similar among groups at all study time points (data not shown). Using 24-h recall, the amount of gas most commonly reported was “slight amount” or “moderate amount” up to 180 days of age and “none at all” or “slight amount” by 275 and 365 days of age. Fussiness was most often characterized as “slightly fussy” or “not at all fussy” in all groups. No significant group differences in mean (±SE) stool frequency (number/day) were detected at any time point assessed (Table 4). No group differences in mean (±SE) stool consistency (with categories corresponding to 1 = hard, 2 = formed, 3 = soft, 4 = unformed or seedy, 5 = watery; Table 4) were detected at baseline. Significant differences in stool consistency were detected between Control and investigational formula groups from day 30 through 180. By category, the primary differences at these study time points were more infants with a formed and fewer infants with an unformed or seedy stool consistency in the Control compared to LF-0.6 and LF-1.0 groups. The majority of infants in all groups from day 30–365 were reported to have a soft stool consistency. There were no significant differences among study formula groups by day 275, possibly reflecting the increased amount of complementary feeding in older children consuming less formula.

**Table 4:** Stool characteristics at 14, 30, 60, 90, 120, 180, 275, and 365 days of age

	Stool frequency			Stool consistency, n (%)					
Age (days)	Group (*n*)	Mean ± SE^a^	Overall *P*	hard	formed	soft	unformed or seedy	watery	Overall *P*
14	Control (154)	3.1 ± 0.2	0.774	2 (1)	9 (6)	75 (49)	62 (41)	5 (3)	0.092
	LF-0.6 (164)	3.3 ± 0.2		0 (0)	4 (3)	71 (45)	77 (48)	7 (4)	
	LF-1.0 (158)	3.3 ± 0.2		2 (1)	6 (4)	77 (49)	65 (42)	6 (4)	
30	Control (133)	2.4 **±** 0.1	0.516	10 (8)	18 (14)	82 (62)	22 (17)	0 (0)	<0.001†‡
	LF-0.6 (152)	2.6 ± 0.1		0 (0)	2 (1)	76 (50)	64 (42)	10 (7)	
	LF-1.0 (141)	2.5 ± 0.1		1 (1)	6 (4)	66 (47)	59 (42)	8 (6)	
60	Control (123)	1.9 ± 0.1	0.661	0 (0)	17 (14)	77 (65)	25 (21)	0 (0)	<0.001†‡
	LF-0.6 (141)	2.0 ± 0.1		0 (0)	1 (1)	77 (57)	51 (38)	7 (5)	
	LF-1.0 (123)	1.9 ± 0.1		0 (0)	1 (1)	65 (55)	45 (38)	8 (7)	
90	Control (118)	2.1 ± 0.1	0.882	2 (2)	11 (9)	79 (68)	23 (20)	2 (2)	<0.001†‡
	LF-0.6 (137)	2.0 ± 0.1		0 (0)	2 (2)	76 (58)	44 (33)	10 (8)	
	LF-1.0 (119)	2.0 ± 0.1		0 (0)	1 (1)	78 (68)	33 (29)	3 (3)	
120	Control (114)	2.0 ± 0.1	0.426	2 (2)	16 (15)	69 (64)	20 (19)	1 (1)	<0.001†‡
	LF-0.6 (135)	1.9 ± 0.1		0 (0)	4 (3)	81 (63)	40 (31)	4 (3)	
	LF-1.0 (113)	1.8 ± 0.1		0 (0)	3 (3)	76 (70)	27 (25)	3 (3)	
180	Control (111)	2.0 ± 0.1	0.242	6 (5)	28 (25)	71 (63)	6 (5)	1 (1)	<0.001†‡
	LF-0.6 (134)	2.3 ± 0.1		1 (1)	18 (14)	91 (70)	20 (15)	0 (0)	
	LF-1.0 (113)	2.1 ± 0.1		1 (1)	8 (7)	88 (79)	15 (13)	0 (0)	
275	Control (108)	2.2 ± 0.1	0.700	5 (5)	36 (33)	62 (57)	5 (5)	1 (1)	0.267
	LF-0.6 (127)	2.1 ± 0.1		3 (2)	35 (28)	78 (62)	5 (4)	4 (3)	
	LF-1.0 (115)	2.3 ± 0.1		5 (4)	26 (23)	74 (65)	6 (5)	2 (2)	
365	Control (94)	2.1 ± 0.1	0.599	6 (6)	33 (35)	47 (51)	6 (6)	1 (1)	0.352
	LF-0.6 (107)	2.2 ± 0.1		4 (4)	29 (27)	65 (60)	9 (8)	1 (1)	
	LF-1.0 (98)	2.0 ± 0.1		4 (4)	36 (38)	47 (49)	2 (2)	7 (7)	

^a^Mean ± standard error (SE) stool frequency

†Control vs. LF-0.6 significantly different (*P <* 0.05)

‡Control vs. LF-1.0 significantly different (*P* < 0.05)

In the overall study population (all participants up to 365 days of age) no statistically significant group differences were detected for study formula discontinuation either related to study formula (Control: 18, 12 %; LF-0.6: 20, 12 %; LF-1.0: 17, 11 %) or not related to study formula (Control: 50, 32 %; LF-0.6: 42, 26 %; LF-1.0: 49, 31 %). Of the 55 participants with formula-related discontinuation, formula intolerance as determined by the study investigator was the most common reason (Control: 13; LF-0.6: 14; LF-1.0: 15) with fussiness (Control: 5; LF-0.6: 8; LF-1.0: 10) and gas (Control: 6; LF-0.6: 3; LF-1.0: 6) as the most common symptoms. Parental decision was the most common reason for discontinuation not related to study formula (Control: 13; LF-0.6: 7; LF-1.0: 19).

No group difference was detected in the number of participants for whom at least one medically-confirmed adverse event was reported (Control: 141, 92 %; LF-0.6: 154, 94 %; LF-1.0: 149; 94 %). There were no statistically significant group differences detected in the overall incidence of adverse events for the following systems: Body as a Whole; Cardiovascular; Eyes, Ear, Nose and Throat; Endocrine; Gastrointestinal (GI); Metabolic and Nutrition; Musculoskeletal; Nervous System; Respiratory; and Skin. Significantly fewer participants in the Control (6, 4 %) and LF-0.6 (7, 4 %) groups versus the LF-1.0 group (17, 11 %; *P* < 0.05) experienced Urogenital system events. Although no significant group differences were detected for specific adverse events in this system, the incidence of penile adhesion (Control: 2, 2 %; LF-0.6: 3, 3 %; LF-1.0: 8, 9 %), commonly associated with circumcision in young male infants, appeared to drive the overall statistical difference. Within the GI System, the medically-confirmed incidence of diarrhea, constipation, emesis, or gas was low with no significant group differences; however gastroesophageal (GE) reflux (Control: 27, 18 %; LF-0.6: 24, 15 %; LF-1.0: 41, 26 %) was significantly lower in the LF-0.6 versus the LF-1.0 group (*P* < 0.05). Within the Nervous System the incidence of macrocephaly (defined as head circumference >98^th^ reference percentile; Control: 3, 2 %; LF-0.6: 0; LF-1.0: 0; *P* < 0.05) was low, albeit statistically significant. No associated underlying health conditions were reported by study investigators for these participants and all completed the study. No group differences were detected in the incidence of allergy- or infection-related adverse events; however, this study was not powered to detect likely subtle differences in this population of healthy term infants. A total of 41 participants (Control: 14, 9 %; LF-0.6: 13, 8 %; LF-1.0: 14, 9 %) experienced serious adverse events (SAEs). With the exception of one SAE in the Control group in which the participant was diagnosed with likely cow’s milk protein intolerance, all SAEs were deemed unrelated to study formulas as assessed by study physicians.

## Discussion

Results of the current study demonstrate routine cow’s milk-based formulas with bLf at 0.6 or 1.0 g/L are associated with normal growth through 365 days of age. With one exception for female weight growth rate in the day 14–60 age range only, no statistically significant group differences were observed for weight, length, or head circumference growth rates from 14–365 days of age. In addition, mean achieved weight for males and females were within the 25^th^ and 75^th^ percentiles of the WHO weight-for-age growth chart from 14–365 days of age and no group differences for mean achieved weight, length, or head circumference were detected. Healthy growth and development have been previously demonstrated in infants receiving bLf [7, 26], however, this is the first large pediatric nutrition trial designed to evaluate growth in which participants received infant formula with concentrations of bLF that correspond to those reported for mature human milk in conjunction with a prebiotic blend of PDX and GOS.

In the current study, acceptance and tolerance of study formulas were good. No differences in overall study discontinuation or study discontinuation due to study formula were detected. No significant group differences were detected in fussiness, gassiness, or mean stool frequency at any measured time point. The majority of infants in all groups from day 30–365 were reported to have a soft stool consistency. However, significant differences in mean stool consistency were detected by day 30 and continued through day 180; more infants with a formed and fewer infants with an unformed or seedy stool consistency in the Control compared to the investigational formula groups were the primary differences. No group differences were observed by day 275, as noted previously, likely reflecting the increased amount of complementary feeding in older children consuming less formula. Collecting a more complete dietary recall after 120 days of age (vs formula intake only) could improve our understanding of the relationship of infant formula and use of complementary foods in feeding practices over the first year of life. Though a current study limitation, at least in evaluation of some secondary outcomes through 365 days of age, this represents a potential next step for future studies.

The primary long-chain polyunsaturated fatty acids found in human milk, DHA and ARA, are always present, albeit at various concentrations and ratios [22, 27, 28]. Previously published values for worldwide human milk DHA and ARA [29, 30] provided the foundation for infant formulas with DHA and ARA (at ~0.3 % and ~0.6 % of total fat, respectively), associated with visual and cognitive development in term infants [31–35] and enhanced growth and neural development in preterm infants [36, 37]. Updated worldwide means for human milk DHA at 0.32 % (SD 0.21 %; median 0.26 %; mode 0.20 %) and ARA at 0.47 % (SD 0.13 %; median 0.45 %; mode 0.50 %) of total fatty acids were published in the most recent comprehensive, critical review of literature [22]. We previously evaluated routine, cow’s milk-based formulas with adjusted ARA and demonstrated they are well-tolerated, safe and promote normal growth from 14–120 days of age [18]. In the current study, routine cow’s milk-based formulas with bLf and adjusted ARA were safe, well-tolerated, and associated with normal growth when fed to healthy term infants through 365 days of age.

The stool softening effect demonstrated with the PDX and GOS prebiotic blend may potentially help address occasional hard stools that could affect formula-fed infants [38]. In healthy, term infants we previously reported that use of routine formulas with a prebiotic blend of PDX and GOS (4 g/L, 1:1 ratio) produced a bifidogenic effect closer to breast milk [17] and softer stools in healthy, term infants compared to formula without PDX and GOS [16–19]. Similarly, Lf may also effect change in the gastrointestinal microbiota. For example, the ratio of fecal *Bifidobacterium* to Enterobacteriaceae and *Clostridium* increased in low-birth weight infants receiving infant formula with bLf (1 g/L) [39] and bLf promoted dose-dependent *Bifidobacterium* growth in vitro [40]. A stronger bifidogenic effect has also been described for pepsin hydrolysates of bLf, suggesting the outcome of gastric digestion on Lf may extend to that found in human milk as well as infant formula with bLf [41]. Based on modified stool consistency observed in this and previous studies, further exploration of potential synergistic effects of providing infant formulas with bLf and the PDX and GOS blend on the infant fecal microbiota and other potentially associated health outcomes may be warranted.

## Conclusions

Routine intact cow’s milk protein infant formulas with bLf at 0.6 and 1.0 g/L were associated with age-appropriate growth throughout the first year of life. This was the first large-scale pediatric nutrition trial in which formulas used concentrations of bLf that are within the range of Lf reported for mature human milk and included the prebiotic blend of PDX and GOS. Compared to infants who received the Control formula, infants who received investigational formulas with the prebiotic blend of PDX and GOS and bLf at 0.6 or 1.0 g/L experienced a softer stooling pattern similar to that reported in breastfed infants. Consequently, this study demonstrated that routine infant formulas with bLf, a blend of PDX and GOS, and adjusted ARA were safe, well-tolerated, and associated with normal growth when fed to healthy term infants throughout the first year of life.

## Abbreviations

AAP: American Academy of Pediatrics
ANOVA: analysis of variance
ARA: arachidonic acid
bLf: bovine lactoferrin
DHA: docosahexaenoic acid
GOS: galactooligosacccharides
Lf: lactoferrin
PDX: polydextrose
